# A Mobilisation Protocol in the Management of Posterior Foetal Presentations: A Multicentre Observational Pilot Study

**DOI:** 10.3390/healthcare14172737

**Published:** 2026-08-27

**Authors:** María Belén Conesa-Ferrer, María José Martínez-Sandoval, Esther María López-Martínez, José Antonio Vera-Pérez, Isabel Baño-Piñero

**Affiliations:** 1 Department of Nursing, Faculty of Nursing, University of Murcia, Av. Buenavista, 32, El Palmar, 30120 Murcia, Spain; javerape@um.es; 2 Vinalopó University Hospital, Carrer Tonico Sansano Mora, 14, Elx, 03293 Alicante, Spain; mariajose.martinezs@um.es; 3 Torrevieja Health Department, Carretera CV-95, Km. 34.400, Partida de la Ceñuela, 03186 Torrevieja, Spain; esthermarialopezmartinez@gmail.com (E.M.L.-M.); bano_isapin@gva.es (I.B.-P.)

**Keywords:** mobilisation protocol, posterior foetal positions, birth, labour, maternal satisfaction, obstetric and neonatal outcomes

## Abstract

**Highlights:**

**What are the main findings?**

- In this pilot observational study (n = 25), anterior rotation was observed in 68% of cases, but in the absence of a control group, these findings cannot be distinguished from spontaneous physiological rotation, and 88% of these achieved vaginal delivery with a 95% confidence interval (76–100%). Apgar scores of ≥9 at 5 min were recorded in 100% of newborns, with an average of 9.96 with a 95% confidence interval (9.88–10.04).
- Women’s level of satisfaction was moderate to high, with the ‘Professional support’ and ‘Participation’ subscales standing out in particular; 100% of the women noted the support they received from the midwife, and 95.24% stated that they had been involved in decision-making.

**What are the implications of the main findings?**

- This protocol for managing labour in cases where the foetus is in an occipito-posterior position could be a very valuable tool for the healthcare professionals attending to these cases.

**Abstract:**

**Background/objectives**: The most common foetal malposition is the occipital posterior (OP) position, which accounts for 33.3% of malpositions occurring during labour, and the rate of dystocia reaches 95%. The aim of this study was to describe observed outcomes of a mobilisation protocol in correcting posterior foetal head positions during labour. **Methods**: A pilot study was conducted using a cross-sectional, multicentre methodology involving 25 pregnant women with a posterior foetal position who were asked to follow a mobilisation protocol. The women’s level of satisfaction was measured using the validated questionnaire, the Childbirth Experience Questionnaire—CEQ (Spanish version). **Results**: Anterior rotation was observed in 68% of cases, but in the absence of a control group, these findings cannot be distinguished from spontaneous physiological rotation, and 88% of these achieved vaginal delivery with a 95% confidence interval (76–100%). Apgar scores of ≥9 at 5 min were recorded in 100% of newborns, with an average of 9.96 with a 95% confidence interval (9.88–10.04). Women’s level of satisfaction was moderate to high, with the ‘Professional support’ and ‘Participation’ subscales standing out in particular; 100% of women noted the support they received from the midwife, and 95.24% stated that they had been involved in decision-making. Regarding perineal outcomes, no third-degree tears were recorded among women who received the protocol, and 44% of participants maintained an intact perineum. **Conclusions**: The protocol was feasible to implement, and anterior rotation, vaginal deliveries, favourable neonatal outcomes, and moderate-to-high maternal satisfaction were observed in this small cohort. However, future research will need to demonstrate the protocol’s efficacy through randomised controlled trials comparing the protocol with standard care.

## 1. Introduction

The most common foetal malposition is the occipital posterior (OP) position, accounting for 33.3% of malpositions occurring during labour [1], with a dystocia rate of 95% [2]. Between 15% and 33% of labours with cephalic presentation present with an OP position; however, most foetuses spontaneously rotate to an anterior position by the end of labour [3]. Around 20–30% of women at the start of the active phase of labour and between 8% and 12% maintain a persistent OP position, defined as the absence of spontaneous rotation of the foetal head [4,5,6,7]. Foetal malposition can be diagnosed by a digital vaginal examination (vaginal examination); this examination has a reliability of 85.7% and is recommended for labour, particularly in delivery units where ultrasound scanning is difficult to perform [8]. Factors associated with foetal malposition include nulliparity, anterior placenta, pelvic shape, use of epidural anaesthesia, increased body mass index, advanced maternal age, and foetal macrosomia [1,9,10,11,12,13].

The OP foetal position is associated with a prolonged second stage of labour and increased pain in the sacral region [14]. It has also been linked to chorioamnionitis, endometritis, severe perineal trauma (third- and fourth-degree perineal tears), higher rates of postpartum haemorrhage, and higher rates of instrumental deliveries and caesarean sections [15,16,17]. With regard to the newborn, foetal injuries at birth have been reported, including brachial plexus injury, foetal hypoxia which may lead to hypoxic-ischaemic encephalopathy, subgaleal haematoma, and an increased risk of requiring neonatal resuscitation and admission to the neonatal intensive care unit [15,16,17,18,19].

Given the morbidities associated with foetal positions, various techniques have been employed to attempt rotation of the foetal head from OP to OA position (occiput anterior position). Manual rotation of an OP foetus in the second stage is widely accepted, with reported success rates of 70% to 90% [20], but the procedure carries risks such as umbilical cord prolapse, cervical laceration, non-reassuring foetal heart rate and foetal injury [21].

Although maternal positioning has been the subject of numerous studies, the evidence regarding its efficacy remains contradictory [22,23]. A recent meta-analysis addressed this issue, concluding that previous research focused on the efficacy of specific positions, whilst its findings emphasise the importance of the details of the intervention (timing of initiation and optimal duration). Furthermore, the synergistic effects of combining different positions or epidural analgesia have not yet been thoroughly studied [24]. Therefore, the aim of the present study was to describe observed outcomes of a mobilisation protocol in correcting posterior foetal head positions during the first and second stages of labour.

## 2. Materials and Methods

### 2.1. Study Design

A pilot study was proposed with a quantitative, observational, cross-sectional, and multicentre methodological approach, carried out in three hospitals in the province of Alicante, from December 2023 to February 2024, involving pregnant women diagnosed with posterior cephalic foetal position. The STROBE guidelines for observational studies were followed in the design of the study (Supplementary Materials).

The diagnosis of posterior foetal position was made through a triple check using Leopold’s manoeuvres (to identify the foetal back), mapping of the maternal abdomen (where the baby’s movements are felt), and a vaginal examination (to identify different structures of the foetal skull). If deemed appropriate or if there were doubts regarding the identification of the foetal position, the obstetrician was asked to perform an ultrasound scan to confirm the position. Furthermore, the signs and symptoms that could indicate the presence of an OP position were as follows [21,22]: a foetal heart rate that is generally heard away from the lateral side of the maternal abdomen; a depression is observed on the maternal abdomen above the pubic symphysis; during Leopold’s manoeuvres, the foetal parts are palpated instead of the foetal back; during the dilation phase, the pregnant woman reports ‘lower back pain’, caused by compression of the sacral nerves by the foetal occiput. This will be a continuous pain that does not subside with contractions; the pregnant woman reports a sensation of bearing down at very early stages; in this context, reference is made to the Ferguson reflex caused by pressure from the foetal occiput and the persistence of the anterior edge of the cervix.

The variables collected were dichotomous qualitative variables: attendance at antenatal classes, employment status, parity, whether or not epidural analgesia was administered, use of oxytocin, degree of foetal head flexion, anterior rotation of the foetal skull, comfort with the positions used in the protocol, and admission of the newborn. The polytomous qualitative variables were nationality, marital status, educational level, type of labour onset, status of the amniotic sac, degree of motor block due to epidural, type of OP position, type of delivery, and condition of the perineum. The variable ‘degree of motor block due to epidural analgesia’ was measured using the Bromage scale, where 0 corresponds to absence of block, 1 for knee mobilisation, 2 for foot mobilisation, and 3 for absence of mobilisation.

The quantitative variables were maternal age (years), number of previous pregnancies, number of previous deliveries, gestational age at delivery (weeks), estimated foetal weight (grams), duration of the active phase of labour until the expulsion stage (hours), number of protocol cycles used, years of experience of the midwife implementing the protocol, Apgar score at one minute and five minutes after the newborn’s birth, total score on the CEQ-E questionnaire, and VAS scales for control, pain, and safety.

#### Outcome Measures

Primary outcome: The single primary outcome was the rate of successful anterior rotation of the foetal skull. This was defined as the proportion of women presenting with a persistent occipito-posterior (OP) position at inclusion who achieved an occipito-anterior (OA) position suitable for spontaneous delivery, assessed at the onset of the active second stage of labour (i.e., at full cervical dilatation, 10 cm, just prior to the expulsive efforts).

Secondary outcomes: The secondary endpoints included the mode of delivery, categorised as normal vaginal, instrumental vaginal and caesarean; maternal satisfaction, measured via the total CEQ-E questionnaire score; perineal trauma, classified according to the degree of laceration (intact perineum, first-degree tear, second-degree tear) and episiotomy; and neonatal adaptation, evaluated by Apgar test scores at 1 and 5 min.

Mobilisation protocol and staff: The foetal head position was initially diagnosed and confirmed at the time of inclusion (active phase of labour) by the attending midwife via transvaginal digital examination, identifying the posterior fontanelle and sagittal suture orientation. A reassessment of foetal head flexion and rotation was systematically performed by the same attending midwife at the moment of full dilatation (10 cm), immediately before the expulsion stage began. All the midwives who took part in the study were highly experienced, with an average of 8.70 years of professional experience (95% confidence interval: 7.17–10.23), and received a standardised training session on the use of the study protocol, in order to ensure inter-observer reliability in the assessment of the primary outcome measure.

Given the interventional, physical nature of the mobilisation protocol (use of specific maternal postures), it was not feasible to blind the midwives applying the protocol or the women themselves.

**Figure 1:**
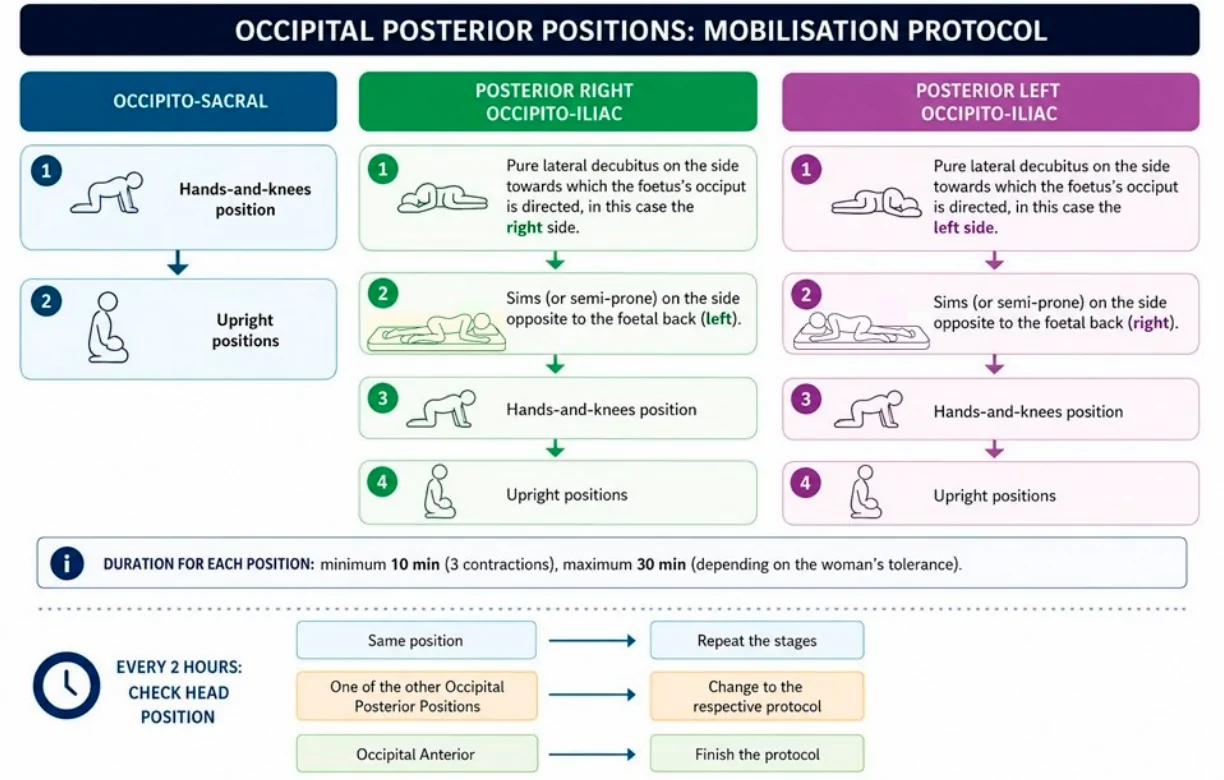
Mobilisation protocol in the management of posterior foetal presentations.

Mobilisation protocol (see Figure 1):

A protocol was designed based on the type of posterior presentation observed. In developing this protocol, various positions were considered that had been shown in previous studies to be effective in rotating the foetus from a posterior to an anterior position [18,22,24,25,26].

Once the woman entered the active phase of labour (4 cm dilated, mature cervix and foetal head at least at Hodge’s plane I), the implementation of the protocols began (each time the full protocol was applied to a woman, we referred to this as a complete cycle; therefore, each woman could undergo several cycles during labour), with the duration of each position recorded. The woman was kept in a position for a minimum of 3 contractions (a time considered effective). The women maintained each position for a minimum of 10 min and a maximum of 30 min. The length of time depended on whether the woman wanted to remain in that position. The degree of foetal head rotation was assessed at least every 2 h during the dilation phase and every 2 h during the expulsion phase [20]. When the woman was examined, the posterior position of the foetal head was identified (occipito-sacral, posterior right occipito-iliac or posterior left occipito-iliac). Depending on the type of position, different postures were applied. If the position was occipito-sacral, the following postures were followed for 2 h or until the next examination, in this order:

1. Hands-and-knees position.
2. Upright positions.

If the presentation was posterior right occipito-iliac, the management protocol was as follows:

1. Pure lateral decubitus on the side towards which the foetus’s occiput is directed, in this case the right side.
2. Sims (or semi-prone) on the side opposite to the foetal back (left).
3. Hands-and-knees position.
4. Upright positions.

If the presentation was posterior left occipito-iliac, the protocol was as follows:

1. Pure lateral decubitus on the side towards which the foetal occiput is directed; in this case, the left side.
2. Sims (or semi-prone) on the side opposite to the foetal back (right).
3. Hands-and-knees position.
4. Upright positions.

The protocol was initiated based on the position of the foetal head observed at that moment, and the same positions were repeated, with a cycle being considered complete once all the described positions had been used. If upon re-examining the woman, the position of the head had changed, the positions corresponding to the baby’s current head position were applied. For example, if the woman began labour with the foetal head in the left OP position, we would start with the pure lateral decubitus position on the side towards which the foetal occiput was directed—in this case, the left side—then right Sims, hands-and-knees position, and finally, vertical positions. Once the woman had been placed in these four positions, a cycle was complete, and the same positions were repeated until the next examination. If, at the next examination, the position had changed to OP sacral, the hands-and-knees position and upright positions were initiated, with a cycle considered complete once both positions had been used.

Each time the mother changed position, she was asked, after the change, whether she had found the position she had adopted comfortable or not.

Experienced midwives implemented the mobilisation protocol.

To measure maternal satisfaction and comfort after childbirth, the validated questionnaire The Childbirth Experience Questionnaire—CEQ (Spanish version) [27] was used, following a request for permission from its authors; it was originally designed to study women’s perceptions of labour and childbirth. This questionnaire has four subscales: ‘Self-efficacy’ (6 items and scales to measure pain level and control), ‘Professional support’ (5 items), ‘Perceived safety’ (5 items and the scale to measure safety level) and ‘Participation’ (3 items). The Spanish version (CEQ-E) has been shown to be a reliable and valid instrument for assessing women’s childbirth experience, with an overall Cronbach’s alpha coefficient of 0.88 [27]. The authors of the CEQ-E satisfaction scale found that the mean CEQ-E scores for the different groups of Spanish women ranged from 2.93 to 3.16 [27]. They calculated these by taking the average of the maximum score on a scale of 1–4 [27]. A total mean score of ≥3.0 was considered to indicate moderate-to-high satisfaction in relative terms. The questionnaire (CEQ-E) was handed out 24 h after delivery and was collected, once completed, before discharge from the maternity unit, 48 h after delivery, regardless of the type of delivery. As there is an emotional bond between the woman and the midwife who attended her during childbirth (because the midwife cared for her during the birth), this does not affect the completion of the questionnaire because the midwife was not present. To minimise any potential social desirability bias arising from the relationship with the care team, a strict data collection procedure was followed: the CEQ questionnaire was self-administered by the women on the maternity ward, with the forms being handed out and collected by nurses who had not been involved in the delivery and in the complete absence of the midwives responsible for the delivery.

The mother’s tolerance of the position, as well as the cardiotocographic readings, were taken into account when adopting the positions; instances where the positions were not applied—due to the woman’s refusal or inability to tolerate them—were recorded.

Once labour was complete, data on obstetric and neonatal outcomes were collected from the electronic medical record: mode of delivery, condition of the perineum, Apgar score, and need for admission to the neonatal unit.

The position of the foetal head was always taken into account when deciding which movements the woman should perform.

### 2.2. Study Population

The population targeted by this study comprised pregnant women with posterior cephalic presentation: OS (occipito-sacral), posterior right occipito-iliac, and posterior left occipito-iliac.

**Figure 2:**
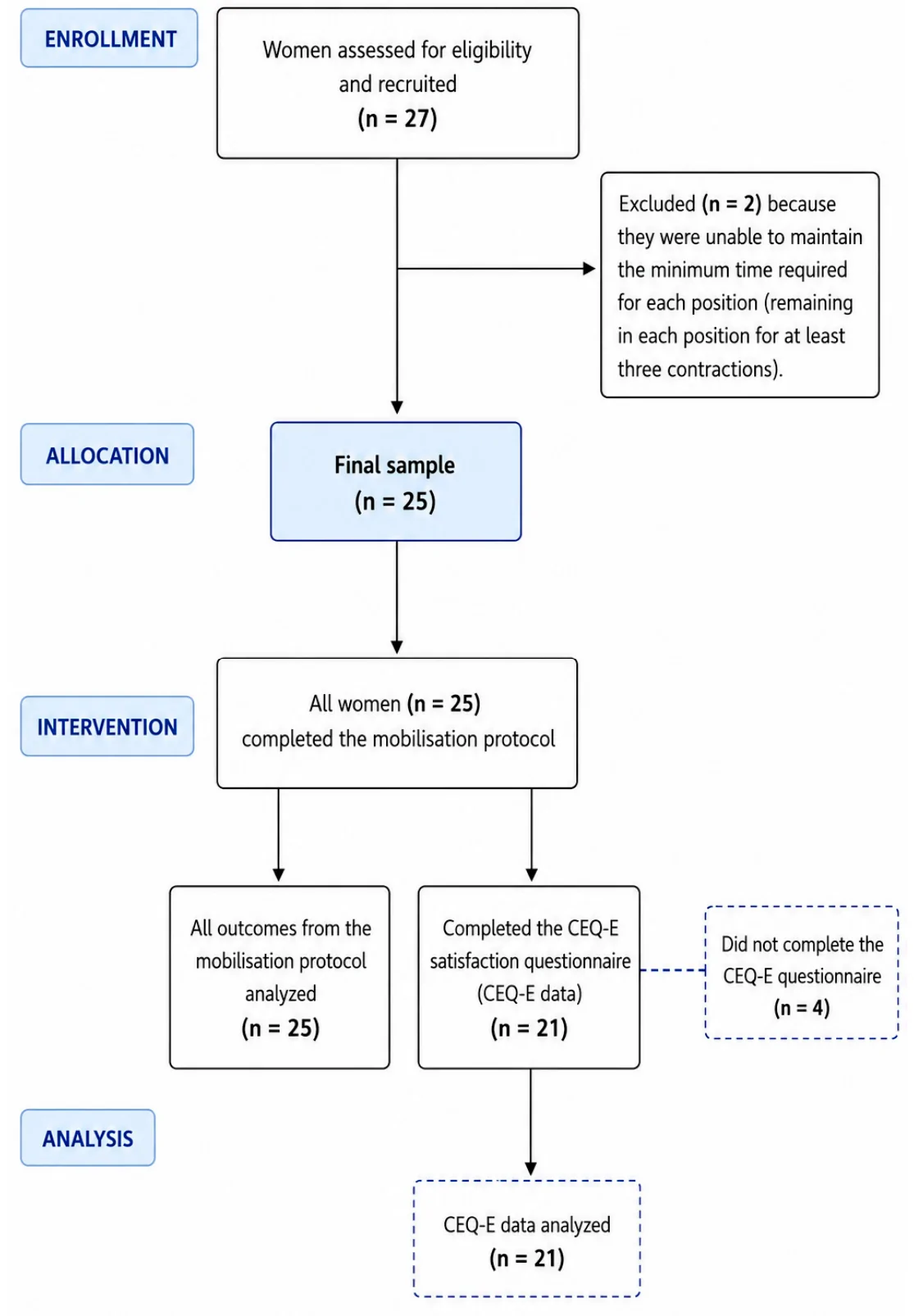
Flow diagram of participant recruitment, intervention, and analysis.

The number of births in the posterior position in the years prior to the study was 5.35% of the total number of births (64.3 births with foetuses in the OP position in each hospital per year). The sample size was determined in accordance with the methodological recommendations for pilot feasibility studies. Given that the primary objective was not to test a hypothesis of efficacy but, rather, to assess the feasibility of the mobilisation protocol and provide preliminary estimates, no conventional statistical power calculation was performed. Our sample size of 25 patients is justified because we based our approach on studies by Totton et al. (2023) which place pilot and feasibility studies within the interquartile range (IQR 20–50), with a median sample size of 30 patients [28]. A sample of 27 women with foetuses in the posterior position was obtained during the 3 months of the pilot study. Two of the women were excluded because they were unable to maintain the minimum time required for each position (maintaining the position for 3 contractions) (see Figure 2). Convenience sampling was used.

The inclusion criteria were full-term pregnant women (37–42 weeks’ gestation) with a singleton cephalic presentation, in the active phase of labour according to WHO criteria (>4 cm dilation), and with a diagnosis of posterior cephalic foetal position.

The exclusion criteria were preterm labour (<37 weeks’ gestation), planned caesarean sections, twin pregnancies, pregnant women with diabetes, pre-eclampsia under treatment, and any condition constituting a contraindication to vaginal delivery (placenta praevia, uterine fibroids).

### 2.3. Ethical Considerations and Data Confidentiality

This project was approved by the ethics committees of the three hospitals where the study was carried out. The names of the hospitals, their identification codes and the dates of approval were: Vinalopó University Hospital, Elche (approval no. 002), on 27 July 2023; Elche General University Hospital (approval no. PI 47/2023), on 28 July 2023; and Torrevieja University Hospital on 15 November 2023. The study was conducted in accordance with the principles of respect, confidentiality and human dignity set out in the Declaration of Helsinki. Furthermore, the Spanish legal and ethical framework was taken into account (Organic Law 3/2018 of 5 December on the Protection of Personal Data and the Guarantee of Digital Rights). Informed consent was obtained from the pregnant women included in the sample who wished to take part in the satisfaction questionnaire. They were informed, via a patient information sheet, about the procedure to be carried out, the preservation of participants’ anonymity, and their right to withdraw from the study at any time.

### 2.4. Data Analysis

Statistical analysis was performed using the SPSS 20.0 statistical software package.

Qualitative variables were analysed using descriptive statistics, including frequency tables and percentages. Quantitative variables were analysed using means, maximum and minimum values, and standard deviation.

Confidence intervals were calculated for the main variables in the study, some at 95% and others at 90%. The confidence interval for quantitative variables was calculated based on the mean. The confidence interval for qualitative variables was calculated on the basis of proportions. Considering the nature of this study as a pilot and exploratory analysis, and with the aim of obtaining estimates of the parameters of interest with a sufficient degree of precision to calculate the sample size for a future trial, a 90% confidence level was chosen for some confidence intervals. This decision, in line with recommendations for feasibility studies, allows for narrower and therefore more informative intervals to be obtained for the planning of subsequent research, provided that the aim of the study is not to draw definitive causal inferences.

The analysis of the CEQ questionnaire was carried out using a listwise deletion approach on the assumption that the pattern of missing responses was completely random.

Due to the nature of the study (pilot study, exploratory in nature), it was not possible to adjust for potential confounders.

## 3. Results

A total of 25 women took part in the study. Of these, 60% were aged between 30 and 36, with 20% under 30 and 20% over 36. The women came from different countries; 68% were from European countries, and 64% were Spanish. There was a higher proportion of first-time mothers (64%) and women who had attended antenatal classes (64%). A total of 40% had a university education, 36% had completed primary education and vocational training, and 24% had no formal education (see Table 1).

With regard to obstetric and neonatal outcomes, the most common OP presentation was posterior right occipito-iliac. None of the women required the position to be confirmed by ultrasound, as the midwives were able to identify it without difficulty by examining the fontanelle and sutures during the vaginal examination. Following the implementation of the protocol, 68% of cases resulted in rotation of the foetal head to the OA position, with a 90% confidence interval of between 53% and 83%; however, in the absence of a control group, these findings cannot be distinguished from spontaneous physiological rotation; 88% of deliveries were vaginal, with a 95% confidence interval of between 76% and 100%, and Apgar scores of ≥9 at 5 min were recorded in 100% of newborns, with an average of 9.96 with a 95% confidence interval (9.88–10.04) (see Table 2 and Table 3).

The quantitative variables are shown in Table 3. With regard to the satisfaction survey, it can be observed that data were missing from the CEQ questionnaire because four women did not complete the satisfaction questionnaire. A complete-case analysis was carried out on the 21 women who completed the questionnaire. The authors of the CEQ-E satisfaction scale considered that a total mean of ≥3.0 indicates that, in relative terms, satisfaction is moderate to high. In our case, applying the same calculation has yielded a result of 3.17. Therefore, satisfaction would be considered moderate to high.

The results of the CEQ-E questionnaire by subscales and items are presented in Table 4. In the ‘Professional Support’ subscale, the high ratings provided by the women to the midwives—who were the professionals who implemented the mobilisation protocol—are noteworthy. These midwives have extensive professional experience, with an average of 8.7 years of service. Also noteworthy are the results of the ‘Participation’ subscale, where 100% of the women stated that they had been able to choose their position during dilation and 95.24% stated that they had been able to choose their position during the expulsion stage, as well as the method of pain relief.

With regard to the comfort experienced by women in the different positions, it was observed that women rated them from most to least comfortable in the following order: left lateral decubitus position, right lateral decubitus position, left Sims, right Sims, upright positions, and quadruped.

## 4. Discussion

The main finding of this study is that the combined mobilisation protocol was associated with foetal rotation into an anterior position. This finding appears to support the view that using a variety of postures may be more helpful than using a single posture [23]. Previous research on maternal mobilisation to correct this malposition, where the methodology was limited to examining a single position and the results indicated that the evidence for its effectiveness was of low certainty or even lacking [23]. The sequential combination of positions, further adjusted according to the lateral position of the occiput, was associated with the observed outcomes in our cohort. These descriptive findings add to the existing literature [24] by providing preliminary data on a dynamic positioning protocol, although they do not constitute confirmatory evidence. Further research in controlled settings is needed to assess the potential clinical applicability of this approach.

Our study recorded a 68% rate of foetal head rotation from OP to anterior following the intervention, but in the absence of a control group, these findings cannot be distinguished from spontaneous physiological rotation. A systematic review by Yang et al., whose analysis shows that maternal positional interventions increase the probability of rotation from OP to OA by 68%, further specifies that a prolonged protocol, starting early at 3–5 cm dilation, doubles the success of rotation and increases spontaneous vaginal delivery by 39% [24]. This latter methodological aspect, which our protocol does indeed fulfil, addresses the gap identified by Barrowclough et al., who emphasise the need to investigate longer and more systematised durations [25]. By maintaining each position for a minimum of three contractions, sufficient exposure time was ensured for the maternal pelvis to facilitate the change in position. In studies using a single position such as the modified Sims position, anterior rotation occurred in 55.1% of cases compared with 21.82% in the control group using free positions [26]. In the randomised clinical trial (RCT) by Wan et al., by combining a sequence of four positions (lateral, modified Sims, quadruped, and genupectoral) for a minimum of 20 min, the percentage of anterior rotation was higher: 66.7% for the group receiving postural intervention alone; for the two groups combining postural intervention with cognitive intervention, the figures were 63.6% and 68.8% versus 27.6% in the control group receiving routine care, which was statistically significant (*p* < 0.05) [29].

Various scientific studies have shown that there is a link between persistent OP presentation and the type of delivery; these studies observed a substantial increase in instrumental deliveries and caesarean sections [9,24,30]. A rate of over 60% of instrumental deliveries and caesarean sections was reported in a study by Falcone et al. [31], and rates of 49.6% of caesarean sections and 31.9% of instrumental deliveries were reported in a study by Eide et al. [32]. Although direct comparisons with studies with a greater amount of evidence are limited by differences in study design, our results show that a rate of 20% in instrumental deliveries and 12% in caesarean sections was recorded, with the majority resulting in spontaneous deliveries (68%). A study by Bueno López et al. on women with persistent OP found that the modified Sims position, when applied consistently, produced a reduction in the caesarean section rate (10.2% in the intervention group vs. 30.9% for the control group) and an increase in the rate of normal deliveries (61.2% in the intervention group vs. 38.1% in the control group) [26]. Regarding the effectiveness of other positions, the systematic review by Yang et al. did show significant differences in the mode of delivery when the positions analysed in separate studies followed a prolonged protocol (a minimum duration or continuous use) starting at 3–5 cm dilation: vaginal delivery and spontaneous vaginal delivery were increased by 22% and 39%, respectively [24]. In line with this, in the study by Wan et al., a significant reduction in the caesarean section rate was achieved compared to the control group in the three groups where a defined mobilisation protocol was used (sequence, in no specific order, of lateral, modified Sims, all-fours, and genupectoral positions; minimum 20 min in each position); these reductions were of 5.7%, 2.9%, and 3.0% versus 20.6% in the control group, *p* < 0.05 [29].

With regard to the duration of labour, although previous research indicates that posterior malposition prolongs both the active and passive phases, it may even slow down the progression of labour [30,31,33]. The average duration observed in our sample was 8.01 h, which is within the range of values reported in the literature. For instance, the RCT by Yang et al. reported a significantly shorter duration of labour in their intervention group, where the Sims’ position was maintained for at least 40 min [16]. Although our observational data align with this trend, we cannot establish a causal relationship between the mobilisation protocol and the duration of labour. Rather, these findings are descriptive and hypothesis-generating, warranting further investigation in controlled settings.

The perineal outcomes observed in this cohort—namely, the absence of third-degree tears and a 44% rate of intact perineum—are noteworthy when considered alongside the existing literature, which identifies the supine position as a risk factor for severe perineal trauma [5,9,15,16,17]. Nevertheless, these findings are descriptive in nature and should not be interpreted as evidence of a protective effect but, rather, as preliminary data warranting confirmation in controlled studies.

It is important to note that in our cohort, 92% of women required oxytocin and the vast majority used epidural analgesia; 64% presented with mild motor block (Bromage grade 1), and the manoeuvres were performed in this context. These findings can be considered alongside those of Lieberman et al. [3] regarding the relationship between epidural anaesthesia and abnormal presentation, although the observational design precludes any causal inference. This pilot study provides initial, hypothesis-generating data suggesting that the structured mobilisation protocol may merit evaluation in future research, including in contexts with high rates of pharmacological intervention. Nevertheless, the observational design precludes any conclusion regarding the protocol’s effectiveness.

Regarding the number of cycles used, the mobilisation protocol in our study was applied with an average of 4.24 cycles per pregnant woman, ranging from one to eight cycles. This sequential and repeated application is consistent with the recommendations of Yang et al., who note that prolonged mobilisation protocols applied continuously are those that significantly increase the rate of spontaneous vaginal delivery and foetal rotation [24]. Furthermore, the use of multiple cycles allows for the investigation of more systematic and prolonged durations, as suggested by Barrowclough et al., to overcome the limitations of previous studies that only analysed isolated or short-duration positions [30].

Regarding neonatal outcomes, our pilot observational data recorded a 5-min Apgar score ≥ 9 in 100% of newborns, with only one case (4%) requiring admission to the neonatal unit. Although the existing literature consistently identifies persistent OP as a risk factor for foetal hypoxia, the need for resuscitation, intensive care admission, and Apgar scores < 7 [15,16,17,18,19,26], our preliminary descriptive findings do not reflect these elevated adverse event rates within this specific small cohort. Nevertheless, given the exploratory nature, limited sample size, and absence of a comparative control group inherent to this pilot design, we interpret these neonatal outcomes with due caution. They should be regarded solely as hypothesis-generating observations that, if replicated in larger, adequately powered prospective studies, could support the neonatal safety of the proposed protocol.

As for the comfort perceived by the pregnant women, it was observed that the positions rated as most comfortable were the lateral recumbent positions (left and right) and the Sims positions, while the upright positions and, in particular, the hands-and-knees position were rated lowest in terms of comfort. These results contrast with other studies in which mothers reported feeling a higher level of comfort in the hands-and-knees position [23]. Although in this study it was observed that women found upright positions less comfortable, other studies have found that women in labour who adopted upright positions experienced faster labour progression, shorter labour duration, and less pain [34]. With regard to lateral positions, other studies conclude that the highest mean comfort scores were obtained when the mother’s lateral position coincided with the alignment of the foetus’s spine during the active phase of labour [35].

Satisfaction with the birth and with professional support was assessed using the CEQ-E questionnaire, yielding moderate-to-high satisfaction scores. Particularly noteworthy is the professional support subscale, where 100% of women stated they ‘strongly agreed’ that they had felt very well cared for by the midwife. These results underscore the importance of the midwife’s role in managing OP malpresentation, which is often associated with negative birth experiences due to lower back pain and a prolonged labour process. Satisfaction is significantly associated with a gentle attitude of the healthcare provider, respect for needs, respect for dignity, and the woman’s inclusion in the decision-making process [36]. Similar results have been found in other studies, which observed that freedom of movement during labour increases maternal satisfaction, as well as the woman’s sense of autonomy [37,38]. Women who used upright positions experienced greater satisfaction than those who adopted reclining positions [34].

This study has several limitations. Firstly, the vast majority of the sample came from European countries (68%) and, in particular, from Spain (64%), which may limit the generalisability of the results to other regions. Furthermore, it should be noted that the small sample size (n = 25) and the fact that the participants were drawn from three hospitals in a single Spanish province limit the generalisability of the results to the entire population. Secondly, the cross-sectional design does not allow for the establishment of causal relationships. This is an observational pilot study; causality cannot be inferred, and the absence of a control or comparison group substantially limits interpretation. The observed 68% rotation rate cannot be attributed solely to the intervention without comparison with standard care. Furthermore, potential reporting bias cannot be ruled out due to the self-reporting nature of the questionnaires and possible cultural interpretations of certain items. As the sample was a convenience sample, selection bias may be present. This approach was necessary due to the limited time available to conduct the study. In addition, as this was a pilot study, the sample size was small, and there was no control group; consequently, no comparisons can be made between groups and the effectiveness of the protocol cannot be established. Further research is therefore needed; a future study should be designed as a randomised clinical trial in which the use of the protocol in the intervention group can be compared with a group receiving standard care.

The fact that the midwives who attended to the women and the women themselves were not blinded to the intervention constitutes a potential source of performance and detection bias. However, the fact that the assessments were carried out by experienced midwives, together with the objectivity of the primary outcome measure—defined by identifiable foetal cranial sutures and fontanelles—mitigates the risk of differential misclassification, and the standardisation of the assessment protocol across all participants reinforces the reliability of our results. In addition, the same midwives who implemented the mobilisation protocol also performed the digital examinations that determined the primary outcome. This lack of blinding introduces a potential risk of detection bias (observer bias), as the clinicians’ expectations regarding the protocol’s effectiveness could theoretically influence their assessment of the foetal position. While our study was not designed to test efficacy but rather to describe outcomes associated with protocol implementation, this limitation precludes any causal interpretation of the observed association between mobilisation and rotation.

Another potential limitation was the possibility of an incorrect classification of the foetal position. The study design initially proposed that two experienced midwives should carry out the vaginal examination to ensure a double-check; however, given the discomfort involved in the procedure and in order to preserve the participants’ privacy as much as possible, it was decided not to do so. Nevertheless, even a double digital examination would not have entirely overcome the inherent subjectivity and inaccuracy of this method. Digital assessment is particularly unreliable in the presence of caput succedaneum, molding, or a high presenting part, and comparative studies have reported discordance between digital and sonographic determination of foetal occiput position in approximately one-fifth to one-third of cases before operative delivery [39]. In this regard, intrapartum sonography has been proposed as a more objective and better-tolerated adjunct to clinical examination. Mappa and Rizzo [40] systematically defined its role by addressing five key questions that digital examination often fails to answer: foetal head position, likelihood of spontaneous rotation in occiput-posterior fetuses (based on foetal spine orientation), flexion/asynclitism (quantified by the occiput-spine angle or chin-to-chest angle), head station (measured by the angle of progression or head-perineum distance), and dynamic descent during pushing. Although these sonographic parameters demonstrate reproducible diagnostic accuracy and consistent prognostic associations with mode of delivery, it is important to note—as highlighted by the same authors [39]—that no adequately powered randomized trial has yet proven that acting upon these measurements reduces maternal or neonatal morbidity. Consequently, the World Association of Perinatal Medicine guidelines endorse ultrasound only as an adjunct when clinical examination is uncertain, not as a routine replacement for digital assessment [40]. Since our study did not incorporate intrapartum sonography, we cannot exclude misclassification bias, particularly regarding unrecognized occiput-posterior positions or deflexion/asynclitism, which may have influenced our reported outcomes. Future studies should evaluate whether implementing the standardized sonographic protocol proposed by Mappa et al. [39] improves clinical decision-making and reduces operative morbidity compared to digital examination alone.

Other limitations include the fact that, due to the nature of the study (a pilot study of an exploratory nature), it was not possible to adjust for potential confounding factors.

Nevertheless, the study has notable strengths; the results observed following the implementation of a personalised mobilisation protocol were very positive for the women and their babies. Furthermore, the use of the CEQ-E, a validated and reliable instrument for assessing the childbirth experience, and its administration just a few hours after birth, reduced the likelihood of recall bias.

## 5. Conclusions

The protocol was feasible to implement, and anterior rotation, vaginal deliveries, favourable neonatal outcomes, and moderate-to-high maternal satisfaction were observed in this small cohort. However, future research will need to demonstrate the protocol’s efficacy through randomised controlled trials comparing the protocol with standard care.

Women’s satisfaction with their birth experience was moderate to high. Women reported feeling involved in decision-making, and the care received from the midwife was also highly rated.

These findings are descriptive in nature and, due to the observational design and lack of a control group, cannot be used to infer efficacy, effectiveness, superiority, or causality. They serve as preliminary, hypothesis-generating data. Future randomised controlled trials are needed to assess whether this protocol offers any advantage over standard practice.

## Figures and Tables

**Table 1:** Sociodemographic data (n = 25).

	N	%
**Ethnicity/nationality**		
Spain	16	64
Other European countries	1	4
South American	5	20
African	3	12
**Marital status**		
Married/Living together	23	92
Single	2	8
Others	0	0
**Education**		
Primary school	6	24
High school	3	12
University	10	40
No studies	6	24
**Parity**		
First-time mothers	16	64
Multiparous	9	36
**Prenatal classes**		
Yes	19	76
No	6	24

**Table 2:** Birth data (n = 25).

	No.	%
**Onset of labour**		
Spontaneous	11	44
Stimulation	3	12
Induction	11	44
**Gestational age (weeks)**		
37	2	8
38	2	8
39	8	32
40	8	32
41	5	20
**Use of oxytocin**		
Yes	23	92
No	2	8
**Degree of motor block due to epidural analgesia**		
Grade 1	7	28
Degree 2	16	64
Degree 3	1	4
Degree 4	1	4
**Occipito-posterior foetal head position**		
Left OP	5	20
Right OP	14	56
OP	6	24
**Mode of delivery**		
Normal vaginal	17	68
Instrumental vaginal	5	20
Caesarean	3	12
**Rotation of the foetal calotte from OP to OA**		
Yes	17	68
No	8	32
**Condition of the perineum**		
Intact perineum	11	44
First-degree tear	2	8
Second-degree tear	8	32
Episiotomy	4	16
**Condition of amniotic sac**		
ARM	7	28
SEM	16	64
IAS	2	8
**Newborn admission**		
Yes	1	4
No	24	96
**Newborn Apgar score**		
One-minute Apgar ≥ 9	18	72
Five-minute Apgar ≥ 9	25	100

ARM (Artificial rupture of membranes), SRM (Spontaneous rupture of membranes), IAS (Intact amniotic sac).

**Table 3:** Quantitative variables.

Quantitative Variables	N	Min	Max	Mean	Std. Dev.
Age (years)	25	23	40	33.00	4.481
Number of pregnancies	25	1	6	1.84	1.344
Previous births	25	0	3	0.64	1.036
Foetal weight (g)	25	2975	4000	3388.00	306.49
Duration from the active phase of labour to the expulsive phase (h)	25	1.61	12.50	8.01	2.92
Number of protocol cycles used	25	1	8	4.24	2.107
The midwife’s experience (y)	20	2	13	8.70	3.262
One-minute Apgar	25	7	9	8.56	0.768
Five-minute Apgar	25	9	10	9.96	0.200
Total CEQ-E score	21	53	66	60.19	3.516
Level of control during childbirth	21	2.20	13.00	8.3143	3.53727
Level of pain during childbirth	21	0.00	12.60	7.4905	3.47274
Level of safety during childbirth	21	6.80	14.00	10.5571	1.86697

**Table 4:** Results of the questionnaire by items of the subscales “Own capacity”, “Professional support”, “Perceived safety” and “Participation”.

Items CEQ-E Questionnaire	TA N(%)	A N(%)	D N(%)	TD N(%)
**Own Capacity**				
The work progressed as I had expected	2(9.52)	9(42.86)	7(33.33)	3(14.28)
I felt strong	4(19.05)	12(57.14)	5(23.81)	0(0)
I felt capable	4(19.05)	9(42.86)	7(33.33)	1(4.76)
I felt tired	0(0)	1(4.76)	12(57.14)	8(38.09)
I felt happy	8(38.09)	7(33.33)	6(28.57)	0(0)
I felt that I handled the situation well	6(28.57)	8(38.09)	6(28.57)	0(0)
**Professional support**				
My midwife devoted enough time to me	17(81)	4(19.05)	0(0)	0(0)
My midwife also devoted enough time to my partner	14(66.7)	7(33.33)	0(0)	0(0)
My midwife kept me informed about what was happening during labour and birth	18(85.6)	3(14.28)	0(0)	0(0)
My midwife understood my needs	18(85.6)	3(14.28)	0(0)	0(0)
I felt very well looked after by the midwife	19(90.5)	2(9.52)	0(0)	0(0)
**Perceived safety**				
I felt scared	2(9.5)	4(19.05)	12(57.14)	3(14.28)
I have many positive memories of the work process	8(38.09)	12(57.14)	1(4.76)	0(0)
I have many negative memories of the labour process	1(4.76)	3(14.28)	10(47.61)	7(33.33)
Some of my memories from the work process make me feel depressed	0(0)	6(28.57)	5(23.81)	10(47.61)
My impression of the medical competence made me feel secure	18(85.6)	2(9.5)	0(0)	1(4.76)
**Participation**				
I felt I could choose whether I should be up and about or lie down	3(14.28)	18(85.6)	0(0)	0(0)
I felt I could choose the delivery position	11(52.38)	9(42.86)	1(4.76)	0(0)
I felt I could choose which pain relief method to use	9(42.86)	11(52.38)	1(4.76)	0(0)

A, agree; D, disagree; TA, totally agree; TD, totally disagree.

## Data Availability

The original contributions presented in this study are included in the article. The data generated in this study appear exclusively in this article; they have not been published in any other source, nor are they available in any other database. For any further enquiries, please contact the corresponding author.

## Supplementary Materials

The following supporting information can be downloaded at: https://www.mdpi.com/article/10.3390/healthcare14172737/s1, STROBE Statement—Checklist of items that should be included in reports of cross-sectional studies.
